# Two Cases of SMARCA4-Deficient Non-small Cell Lung Cancer (NSCLC) with Improved Performance Status (PS) after Treatment with Immune Checkpoint Inhibitors (ICIs)

**DOI:** 10.7759/cureus.37656

**Published:** 2023-04-16

**Authors:** Akiko Koizumi, Yukiho Tamura, Ryohei Yoshida, Chie Mori, Yusuke Ono, Mishie Tanino, Yusuke Mizukami, Takaaki Sasaki

**Affiliations:** 1 Medicine, Asahikawa Medical University School of Medicine, Asahikawa, JPN; 2 Respiratory Center, Asahikawa Medical University Hospital, Asahikawa, JPN; 3 Biomedical Research, Sapporo Higashi Tokushukai Hospital, Sapporo, JPN; 4 Diagnostic Pathology, Asahikawa Medical University Hospital, Asahikawa, JPN; 5 Gastroenterology and Hematology/Oncology, Department of Medicine, Asahikawa Medical University, Asahikawa, Hokkaido, JPN

**Keywords:** brg1, performance status, immune checkpoint inhibitor, non-small cell lung cancer, smarca4-deficient

## Abstract

SWItch/Sucrose Non-Fermentable (SWI/SNF)-related, matrix-associated, actin-dependent regulator of chromatin, subfamily A, member 4 (SMARCA4) mutations are commonly reported in non-small cell lung cancer (NSCLC) and are associated with a poor prognosis. There is insufficient evidence regarding the efficacy of immune checkpoint inhibitors (ICIs) in SMARCA4-deficient NSCLC patients with poor performance status (PS). We report two cases of advanced SMARCA4-deficient NSCLC treated with ICIs, in which marked regression of the tumor and improved general condition of the patients were achieved.

## Introduction

SWItch/Sucrose Non-Fermentable (SWI/SNF)-related, matrix-associated, actin-dependent regulator of chromatin, subfamily A, member 4 (SMARCA4)-deficiency caused by SMARCA4 gene mutations are reported in 5-10% of all non-small cell lung cancer (NSCLC) cases [1-3].

Although there have been recent reports that immune checkpoint inhibitors (ICIs) are effective in SMARCA4-deficient lung cancer, there is currently no reliable opinion regarding ICI treatment for patients in poor general condition, specifically those with a European Cooperative Oncology Group (ECOG) performance status (PS) of 3 or 4. ICIs are not recommended in the 2021 Japanese Lung Cancer Society Guidelines for Diagnosis and Treatment of Lung Cancer due to lack of evidence. To the best of our knowledge, no previous reports have focused on improvement of PS in SMARCA4-deficient NSCLC patients. Thus, we report two cases of advanced SMARCA4-deficient NSCLC treated with ICIs that demonstrated regression of the tumor and improved general condition of the patients.

## Case presentation

Case 1

**Figure 1:**
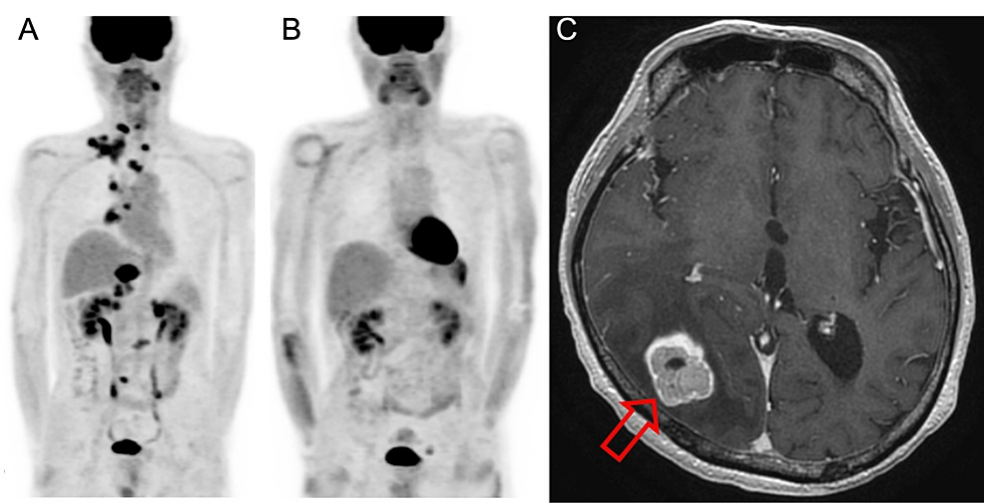
Case 1 (A) 18F-fluoro-D-deoxyglucose (FDG) positron emission tomography (PET)/CT before pembrolizumab treatment shows FDG uptake in the lower lobe of the right lung, multiple lymph nodes, multiple bone sites, and bilateral adrenal glands; (B) PET-CT after 48 cycles of pembrolizumab shows loss of uptake in all lesions; (C) Brain MRI after discontinuation of pembrolizumab shows a single brain metastasis in the right occipital lobe as indicated by the red arrow.

A 63-year-old man, a former smoker with no other medical history, presented with hoarseness and swelling of the right neck. CT showed enlarged mediastinal and right supraclavicular lymph nodes and a nodular lesion in the right lower lobe of the lung. 18F-fluoro-D-deoxyglucose positron emission tomography (FDG-PET) showed multiple lymph nodes and liver and bone metastases (Figure 1). A biopsy of the right supraclavicular lymph node was performed, and the diagnosis was poorly differentiated NSCLC, cT4N3M1b cStage IVB.

A cisplatin + pemetrexed + bevacizumab regimen was started as first-line treatment, and the response was stable disease (SD) according to Response Evaluation Criteria in Solid Tumors (RECIST) guidelines. After three cycles, renal dysfunction occurred, and chemotherapy was discontinued. Seven months after the start of first-line treatment, the patient complained of worsening fatigue and loss of appetite, and he visited the emergency department with severe dehydration and renal dysfunction (blood urea nitrogen (BUN): 52.3 mg/dL, Creatinine: 2.73 mg/dL, estimated glomerular filtration rate (eGFR): 20.2 mL/min) and was immediately hospitalized. FDG-PET showed progression of multiple lymph node, bone, and liver metastases, and new metastases in the adrenal glands (Figure 1A). Since the patient had worsening PS with a score of 2-3, cytotoxic chemotherapy was deemed high risk. Palliative radiation therapy was performed for lumbar spine metastasis.

**Figure 2:**
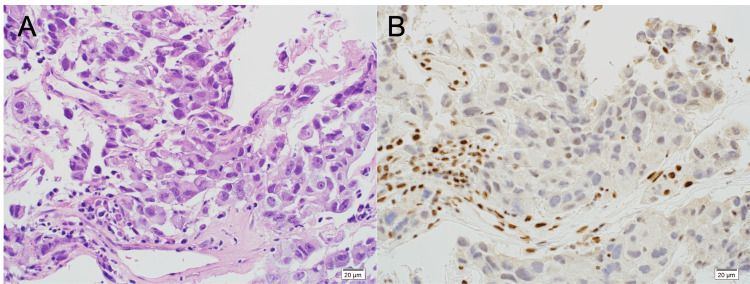
Case 1—Histopathological findings of the brain metastasis specimen (A) Hematoxylin and eosin (H&E) staining; (B) Immunohistochemical staining for BRG1

Biopsy of the lower right lung lesion confirmed high expression (50-60%) of PD-L1 (22C3), and second-line pembrolizumab was started. Five months later, a psoriasiform rash developed on the left elbow, but was grade 2 according to Common Terminology Criteria for Adverse Events (CTCAE); therefore, pembrolizumab was continued. The cancer regressed, achieving RECIST complete response (CR). The patient’s PS also improved to 0. Pembrolizumab was continued for 48 cycles (Figure 1B) then discontinued according to the patient’s wishes. Nine months later, the patient had a seizure, and a single brain metastasis was found on MRI (Figure 1C). Surgical excision of the brain metastasis was performed. Although the multiplex Oncomine® Dx Target Test for mutations in 46 driver-genes was negative, immunohistochemistry showed SMARCA4/BRG1 nucleic staining to be negative (Figure 2). Upon resuming pembrolizumab treatment, no relapses were observed; the patient's follow-up survival period after the initial dose of pembrolizumab has extended to over four years.

Case 2

A 54-year-old man, a former smoker, was found to have a large mass in the left upper lung field on chest radiography and was immediately referred to a university-affiliated hospital. CT showed severe emphysema, giant bullae, a 10-cm tumor in the left upper lobe of the lung, left pleural effusion, enlarged mediastinal and abdominal lymph nodes, and enlarged adrenal glands. CT-guided biopsy of the lung tumor confirmed adenocarcinoma. Staging with FDG-PET was cT4N3M1c cStage IVB. During the three weeks while waiting for definitive diagnosis and driver-gene analysis, the patient’s PS worsened drastically due to quick progression of the cancer, causing hypoxia and worsening back pain. At this point, he was referred to our hospital for treatment.

**Figure 3:**
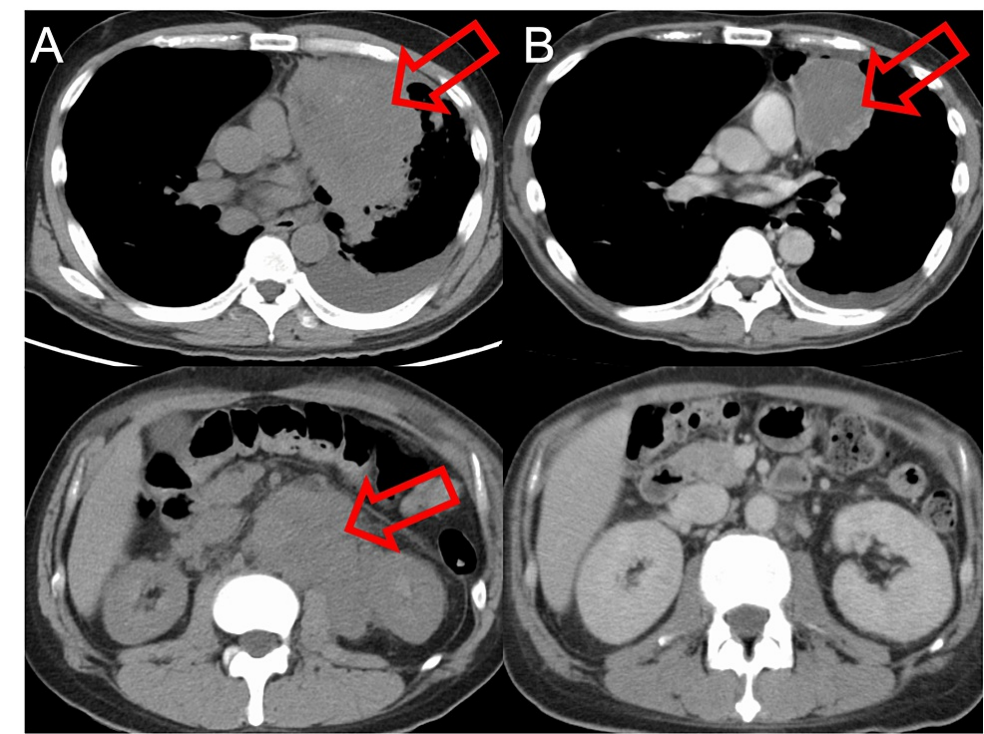
Case 2 (A) Before treatment, chest CT showed a 10-cm tumor in the left upper lobe of the lung (indicated by red arrow) and a left pleural effusion (upper), and abdominal CT showed a large tumor clustered together with lymph nodes (indicated by red arrow) (lower); (B) Two months after starting ICIs in combination with cytotoxic chemotherapy, chest CT showed shrinkage of the left upper lobe tumor (indicated by red arrow) and reduction of left pleural effusion (upper), and abdominal CT showed marked reduction of the abdominal tumor (lower). ICI: Immune checkpoint inhibitors

The patient was admitted and immediately given continuous intravenous opioids. Although the results for PD-L1 and driver-gene analysis had not yet been confirmed, the extremely aggressive clinical course called for immediate treatment. His PS score was 4, life expectancy was less than three months, and chemotherapy was deemed high risk. After thorough explanation of treatment risk, the patient requested treatment, and a carboplatin + paclitaxel + pembrolizumab regimen was started as first-line treatment. After one cycle, the thoracic tumors decreased in size. On the other hand, the peritoneal lymph nodes became enlarged. The following month, the treatment regimen was changed to carboplatin + paclitaxel + ipilimumab + nivolumab as second-line treatment, resulting in a marked reduction of tumor size (Figure 3), and an improvement of the PS score to 1.

**Figure 4:**
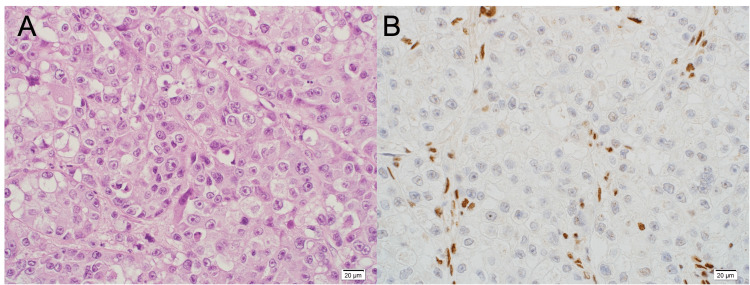
Case 2—Histopathological findings of the lung specimen (A) Hematoxylin and eosin (H&E) staining; (B) Immunohistochemical staining for BRG1

Histopathology confirmed the PD-L1 (22C3) tumor proportion score (TPS) to be 5%. The Multiplex Oncomine® Dx Target Test detected no mutations. Immunohistochemistry confirmed a loss of SMARCA4/BRG1 nuclear staining (Figure 4). Six weeks after switching to second-line treatment, the patient developed serum creatinine elevation of CTCAE grade 2 along with enlargement of the kidneys on CT, and this was diagnosed as renal dysfunction as an immune-related adverse event (irAE). Treatment with oral prednisolone 1 mg/kg (60 mg) per day was started, and renal function subsequently improved. Since then, prednisolone has been tapered to 5 mg/day. The patient was switched to nivolumab monotherapy, and during our follow-up, which covers at least 6 months from the initiation of treatment, has consistently maintained a RECIST partial response (PR).

## Discussion

SMARCA4 is the gene encoding the protein BRG1, a core subunit of the SWI/SNF chromatin remodeling complex, which has enzymatic properties to alter chromatin structure so that DNA becomes accessible for transcription, replication, and repair. SMARCA4 is also known to function as a tumor suppressor gene [4]. In 2015, Loarer et al. were the first to report a case of SMARCA4-deficient thoracic sarcoma [5]. Since then, it has been reported that SMARCA4 deficiency is commonly observed in NSCLC, and has been gaining attention as a unique subset of lung cancer [1-3]. SMARCA4-deficient NSCLC is known to occur at a relatively young age, is more common in smokers, has a poor prognosis, and is usually negative for TTF-1 on immunohistochemical staining [3,6-9].

The treatment efficacy of ICIs can be predicted by tumor cell expression of PD-L1. In Case 1, PD-L1 expression was high, with a TPS of 50-60%; therefore, ICI monotherapy was predicted to be effective. In the KEYNOTE-010 clinical trial, pembrolizumab monotherapy was shown to be effective in previously-treated advanced or recurrent non-operable NSCLC with PD-L1 TPS ≥1%. Patients with high PD-L1 expression (TPS ≥50%) had a median OS of 14.9 months (2 mg/kg group) and 17.3 months (10 mg/kg group) [10]. However, the present Case 1 has achieved much longer survival. Integrated analysis of several clinical studies demonstrated that, regardless of PD-L1 expression, CR rate achieved by ICIs is only 1.5% [11]. Therefore, it can be said to be very rare to achieve CR despite multiple metastases, as in Case 1. In the Checkmate 9LA study, nivolumab and ipilimumab in combination with cytotoxic chemotherapy demonstrated a median OS of 15.8 months and a response rate (PR or above) of 39.3% (50/127) in NSCLC with PD-L1 TPS 1-49% [12]. Considering these results, it can be said that Case 2 responded well to ICIs, although the observation period is currently less than 2 years and needs further evaluation.

Predictors of ICI benefit other than PD-L1 expression are pre-treatment tumor size and number of metastases [13]. In Case 1, there were a total of four distant metastases before treatment with pembrolizumab. In Case 2, pre-treatment primary tumor size was greater than 10 cm. We hypothesize that there were factors other than PD-L1 that accounted for the marked tumor regression in both of the present cases. In NSCLC, co-mutations such as STK11* *and KEAP1 are known to cause resistance to ICI therapy. There are contradictory reports of whether SMARCA4 also causes resistance to ICI treatment [3,6,14].

There are still conflicting ideas regarding treatment for patients with poor general condition due to cancer progression. The 2021 Japanese Lung Cancer Society Guidelines for Diagnosis and Treatment of Lung Cancer do not currently support the use of ICIs in patients with a low PS score. The study by Rizzo et al. highlights that a decline in PS correlates with decreased progression-free survival (PFS) and overall survival (OS) in patients with a PS of up to 2 [15]. Nevertheless, the research does not conduct an extensive analysis of PS concerning specific mutations, such as SMARCA4. Conversely, there are numerous reports of cases where ICIs have resulted in substantial regression of severely advanced cancer, unattainable through traditional chemotherapy [16]. As illustrated in the case report, there are examples of remarkable recoveries in SMARCA4-deficient cases, indicating that further exploration is merited.There is a possibility that ICIs may be effective in the treatment of relatively young, progressive NSCLC, especially in the absence of SMARCA4/BRG1, but further case series and clinical trials are needed to confirm this possibility.

## Conclusions

Our experience with two cases of SMARCA4-deficient NSCLC patients highlights the potential of ICIs to improve PS in this specific population. The findings suggest that, particularly for younger patients with rapidly progressive NSCLC, ICIs may serve as a valuable therapeutic option even in the presence of poor PS when SMARCA4 deficiency is identified. Further investigation is warranted to confirm the broader applicability and efficacy of this promising treatment approach.

## References

[1] Araujo LH, Timmers C, Bell EH (2015). Genomic characterization of non-small-cell lung cancer in African Americans by targeted massively parallel sequencing. J Clin Oncol.

[2] Dagogo-Jack I, Schrock AB, Kem M (2020). Clinicopathologic characteristics of BRG1-deficient NSCLC. J Thorac Oncol.

[3] Schoenfeld AJ, Bandlamudi C, Lavery JA (2020). The genomic landscape of SMARCA4 alterations and associations with outcomes in patients with lung cancer. Clin Cancer Res.

[4] Mittal P, Roberts CW (2020). The SWI/SNF complex in cancer - biology, biomarkers and therapy. Nat Rev Clin Oncol.

[5] Le Loarer F, Watson S, Pierron G (2015). SMARCA4 inactivation defines a group of undifferentiated thoracic malignancies transcriptionally related to BAF-deficient sarcomas. Nat Genet.

[6] Alessi JV, Ricciuti B, Spurr LF (2021). SMARCA4 and Other SWItch/sucrose non-fermentable family genomic alterations in NSCLC: clinicopathologic characteristics and outcomes to immune checkpoint inhibition. J Thorac Oncol.

[7] Velut Y, Decroix E, Blons H (2022). SMARCA4-deficient lung carcinoma is an aggressive tumor highly infiltrated by FOXP3+ cells and neutrophils. Lung Cancer.

[8] Naito T, Udagawa H, Umemura S (2019). Non-small cell lung cancer with loss of expression of the SWI/SNF complex is associated with aggressive clinicopathological features, PD-L1-positive status, and high tumor mutation burden. Lung Cancer.

[9] Reisman DN, Sciarrotta J, Wang W, Funkhouser WK, Weissman BE (2003). Loss of BRG1/BRM in human lung cancer cell lines and primary lung cancers: correlation with poor prognosis. Cancer Res.

[10] Herbst RS, Garon EB, Kim DW (2021). Five year survival update from KEYNOTE-010: pembrolizumab versus docetaxel for previously treated, programmed death-ligand 1-positive advanced NSCLC. J Thorac Oncol.

[11] Li J, He Q, Yu X, Khan K, Weng X, Guan M (2019). Complete response associated with immune checkpoint inhibitors in advanced non-small-cell lung cancer: a meta-analysis of nine randomized controlled trials. Cancer Manag Res.

[12] Paz-Ares L, Ciuleanu TE, Cobo M (2021). First-line nivolumab plus ipilimumab combined with two cycles of chemotherapy in patients with non-small-cell lung cancer (CheckMate 9LA): an international, randomised, open-label, phase 3 trial. Lancet Oncol.

[13] Schakenraad A, Hashemi S, Twisk J (2021). The effect of tumor size and metastatic extent on the efficacy of first line pembrolizumab monotherapy in patients with high PD-L1 expressing advanced NSCLC tumors. Lung Cancer.

[14] Marinelli D, Mazzotta M, Scalera S (2020). KEAP1-driven co-mutations in lung adenocarcinoma unresponsive to immunotherapy despite high tumor mutational burden. Ann Oncol.

[15] Rizzo MM, Bluthgen MV, Recondo G (2021). Outcomes of patients with non-small cell lung cancer and poor performance status treated with immune checkpoint inhibitors in the real-world setting. Int J Clin Oncol.

[16] Pluvy J, Brosseau S, Naltet C (2017). Lazarus syndrome in nonsmall cell lung cancer patients with poor performance status and major leukocytosis following nivolumab treatment. Eur Respir J.

